# Lane Departure Warning Mechanism of Limited False Alarm Rate Using Extreme Learning Residual Network and ϵ-Greedy LSTM

**DOI:** 10.3390/s20030644

**Published:** 2020-01-23

**Authors:** Qiaoming Gao, Huijun Yin, Weiwei Zhang

**Affiliations:** 1 The School of Mechanical and Transportation Engineering of Guangxi University of Science and Technology, Guangxi 545005, China; 2 Shanghai University of Engineering Science, Shanghai 201620, China; Weiweiz@sues.edu.cn

**Keywords:** lane departure warning mechanism, false alarm rate, Extreme Learning Residual Network, ϵ-greedy LSTM

## Abstract

Neglecting the driver behavioral model in lane-departure-warning systems has taken over as the primary reason for false warnings in human–machine interfaces. We propose a machine learning-based mechanism to identify drivers’ unintended lane-departure behaviors, and simultaneously predict the possibility of driver proactive correction after slight departure. First, a deep residual network for driving state feature extraction is established by combining time series sensor data and three serial ReLU residual modules. Based on this feature network, online extreme learning machine is organized to identify a driver’s behavior intention, such as unconscious lane-departure and intentional lane-changing. Once the system senses unconscious lane-departure before crossing the outermost warning boundary, the ϵ-greedy LSTM module in shadow mode is roused to verify the chances of driving the vehicle back to the original lane. Only those unconscious lane-departures with no drivers’ proactive correction behavior are transferred into the warning module, guaranteeing that the system has a limited false alarm rate. In addition, naturalistic driving data of twenty-one drivers are collected to validate the system performance. Compared with the basic time-to-line-crossing (TLC) method and the TLC-DSPLS method, the proposed warning mechanism shows a large-scale reduction of 12.9% on false alarm rate while maintaining the competitive accuracy rate of about 98.8%.

## 1. Introduction

### 1.1. Motivation

**Figure 1:**
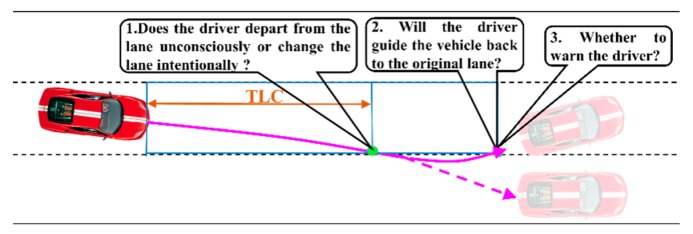
Identification process of driver’s unconscious lane departure behavior.

According to the National Highway Traffic Safety Administration (NHTSA), lane departure is responsible for about 50% of vehicle collision accidents approximately. Recent impressive lane-departure-warning systems are capable of preventing these accidents’ occurrences [1,2]. However, most current lane-departure-warning systems only depend on the relative distance between the vehicle and the road edge to trigger warning signals [3,4,5,6], and are vulnerable to a high false alarm rate for drivers’ intentional lane departure behaviours in the real-world environment, provoking drivers’ boredom and impatience. As shown in Figure 1, the lane departure warning mechanism (LDWM) ought to precisely identify the driver’s intention and give a driver an appropriate warning [7]. A successful LDWM need to concentrate on offering precise assistance and, meanwhile, allocating attention to understanding the driving intention of drivers.

### 1.2. Related Work

The traditional LDWM use TLC [7] to decide whether to touch off a warning. However, this algorithm’s performance is primarily dependent on the setting of the alarm threshold, so it cannot judge the driver’s unconscious lane-departure behaviours (ULDB), causing an excessive false alarm rate (FAR). Yet drivers usually make intentional lane-changing behaviours (ILCB), or proactive correction after slight departure (PCSD), as shown by the solid purple in Figure 1. A warning is activated when the driver unconsciously moves away from the lane boundary and will not go back to the original lane. Therefore, it is crucial to discriminate the initial episode of lane-departure behaviours, judge whether the driver could manipulate the vehicle to return to the original lane, and then determine when the driver should be warned, so as to reduce excessive false warning. 

Misunderstanding of ILCB is the main reason for high false warnings of LDWM. Many experts have studied the identification of driver’s intention to change the lane. Agamennoni et al. [8] introduced an unsupervised method to automatically detect boundaries between driving manoeuvres. However, this pipeline depends on a key assumption that all the data follows a linear Gaussian law within each segment, which cannot be guaranteed when applied to naturalistic lane-change events. Furthermore, Satzoda et al. [9] demonstrated an overall naturalistic driving study hierarchy, which combines the lower-level sensor fusion for all these data, as well as higher-level driving event recognition and driver behaviour analysis. Franziska Bocklisch [10] proposed a new fuzzy system using adaptive fuzzy pattern classification (AFPC) for data-based online evolvement identifying the driver’s lane change intention. Mc Call J C [11] used a sparse Bayesian learning method to judge the intention of driver’s lane change in combination with lane location information, vehicle parameters, and driver’s head motion. Although these aforementioned methods could obtain satisfied classification performance of driver’s intention to change the lane or depart from the lane, they not could mine deeper into sensor data information (e.g., steering wheel angle and lateral acceleration). Recently, time series classification has been considered as one of the most representative methods in data mining. Zhiguang Wang et al. [12] compared the performance of three networks of multilayer perceptron (MLP), fully convolutional networks (FCN), and the residual networks (ResNet) in multivariate time series classification. MLP has poor transferability, and the amount of its weight is directly dependent on the length of the input variable; both FCN and ResNet can pre-train the model on the source dataset and then transmit and fine-tune the model on the target dataset without modifying the hidden layer of the network [13]. ResNet can solve the degradation caused by increasing the network depth relative to FCN [14,15]. Guang-Bin Huang et al. [16] found that Extreme Learning Machine (ELM) can classify any disjoint areas. ELM is a fast learning method for calculating the network weight between the output and hidden layers in a single iteration, which can dramatically reduce learning time while generating accurate results from the least training data [17].

In additional, many advanced warning techniques combined with driver correction behaviour (DCB) have been proposed to reduce FAR. DCB causes the driver to guide the vehicle return to the centre before going near the lane boundary. P. Angkititrakul et al. [18] used the driver behaviour model of segmented lateral slope direction sequence to identify lane-crossing events and driver correction events. Experiment results showed that the algorithm obtained a 17% FAR in detecting an intentional correction when the prediction time was 0.5 s. Although this approach can improve FAR by identifying DCB, it ignored the driver’s other correction behaviours, called PCSD. In the real world, it is normal for a driver to pass through the lane boundary by 0.1–0.2m. Considering PCSD, the concept of virtual lane boundary was proposed [19,20]. Lane departure warning systems based on the virtual lane boundary took driver’s driving habits and road conditions into account, allowing the driver to cross the actual lane boundary in a certain extent. The number of alarms from this algorithm is high for drivers who tend to drift a lot. For the driver proactive correction behaviour prediction, Wenshuo Wang et al. [21] utilized the Gaussian mixture model and the hidden Markov model to predict vehicle trajectory, which was used to judge if the driver will guide the vehicle back to the lane. Dongkui Tan utilized Deep Fourier Neural Network (DFNN) to predict imminent maximum lateral offset, which was used to judge if driver correction event will occur [22]. However, vehicle sensor data is dynamic and changes over time. The prediction of the driver’s future behaviour is essentially a time-series prediction problem. With the development of the machine learning method, long short term memory (LSTM), in particular, has achieved good performance in the time series prediction problem [23,24]. For example, Jae Young Choi et al. [25] tested four challenging time series and showed that LSTM more effectively captured the dynamic behaviour of real-world time series than other famous prediction algorithms. Hua, Y et al. [26] compared improved LSTM with three popular prediction methods: SVR, ARIMA, and FFNN, and found improved LSTM had lower computing costs and satisfactory performance. 

### 1.3. Contributions

In this paper, we proposed a machine-learning-based classification and prediction algorithm to identify ILCB and PCSD respectively. This paper has the following contributions:

(1) We applied a new learning framework termed Extreme Learning Residual Network (ELR-Net) that combines ResNet and ELM to classify drivers’ lane-departure consciousness (ILCB or ULDB). The driver’s intention to change the lane can be accurately identified at 1.3 seconds before the vehicle crosses the lane marking [27]. ELR-Net can accurately determine the driver’s intention to change lanes.
(2) We developed ϵ-greedy-based long short-term memory (ϵ-greedy LSTM) module to forecast the vehicle’s upcoming lateral distance to infer the chance of PCSD. ϵ-greedy LSTM can accurately predict driver’s departure intention.
(3) We correspondingly proposed an LDWM to whether a warning should be given to the driver based on the algorithm of classification and prediction.

## 2. Lane Departure Warning Mechanism

**Figure 2:**
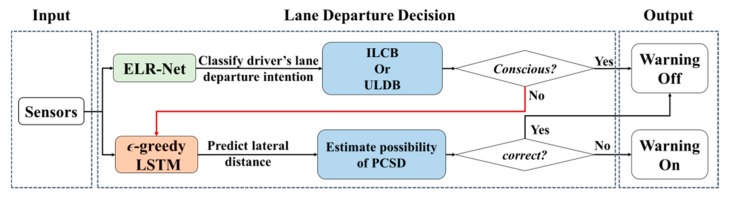
The structure of the proposed lane departure warning mechanism (LDWM).

LDWM aims to judge whether an unconscious lane-departure behaviour will occur, allowing a driver to take effective action and prevent a crash. In this paper, the structure of the proposed LDWM is organized as shown in Figure 2. First, ELR-Net is employed to distinguish between ILCB and ULDB. When the lane departure state is defined to be unconsciously crossing the lane, ϵ-greedy LSTM is used to consecutively predict the forthcoming lateral distance to estimate the probability of PCSD. The process of lane-departure behaviour classification is based on the TLC time interval, as shown in Figure 1. A warning is triggered when the driver unconsciously moves away from the lane boundary and will not return to the original lane in a short period of time. 

### 2.1. Selection of Sensor Input Parameters

Obviously, taking all the relevant factors such as driver-vehicle-road characteristic into account is beneficial to obtaining reliable inference in a real-world environment [28]. The parameters about steering wheel angle α and its angular velocity $\omega_{\alpha }$ represent driver’s operation intention, lateral acceleration $a_{y}$ and yaw rate $\omega_{r}$ are selected to analyse the moving state of the vehicle, and lateral distance D and transverse average velocity ${\bar{v}}_{y}$ are direct measurements of the dynamic distance relationship between vehicle and lane marking. Lateral distance D is defined as the distance from the vehicle’s left (right) front tire to the left (right) lane edge. Here, the six parameters are chosen to analyse the driver’s dynamic intention and combined into one vector, which is defined as $I_{DVR}$ and given by

(1) $$I_{DVR}=\left[\alpha ,\omega_{\alpha },a_{y},\omega_{r},D,{\bar{v}}_{y}\right]$$

### 2.2. Time to Lane Crossing (TLC)

**Figure 3:**
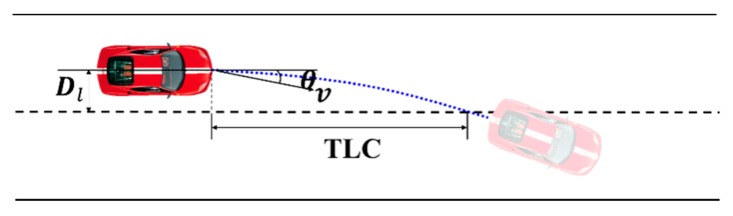
Illustration of time-to-line-crossing (TLC). Black dotted lines and solid lines represent lane lines, and the blue dotted line is vehicle trajectory.

TLC [7] is the time required for the vehicle to cross the lane boundary as shown in Figure 3, and the calculation formula is as follows:

(2) $$TLC=D_{l}/v\sin\theta$$

where $D_{l}$ is the transverse distance between the host car and the lane line, v is the vehicle speed, and θ is the heading angle of the vehicle relative to the lane.

### 2.3. Excessive False Alarm Rate

**Figure 4:**
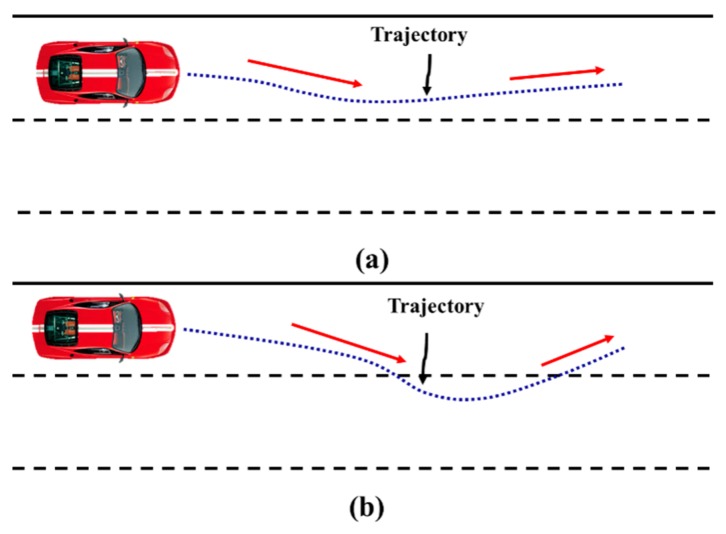
Vehicle trajectory of (**a**) driver correction behavior (DCB) and (**b**) proactive correction after slight departure (PCSD).

Misunderstanding of ILCB is the primary reason for false alarm rate of LDWM. Another reason for the excessive false warning is that some drivers will control the vehicle back to the centre of the lane without the help of a warning. Figure 4 shows two cases consisting of DCB and PCSD. Many studies have considered DCB in Figure 4a [18,19,20,21,22], however, a lane departure warning is also not desired in Figure 4b, because some drivers habitually drift a little beyond lane boundary during driving and then return to the original lane. Therefore, to reduce false alarm rate, we need to judge the driver’s behaviour by asking, “Is the driver about to change the lane or depart from the lane?” and “will the driver guide the vehicle return to the original lane without the help of a warning in a short span of time after the vehicle crosses the lane?”.

### 2.4. Evaluation Criteria for PCSD

**Figure 5:**
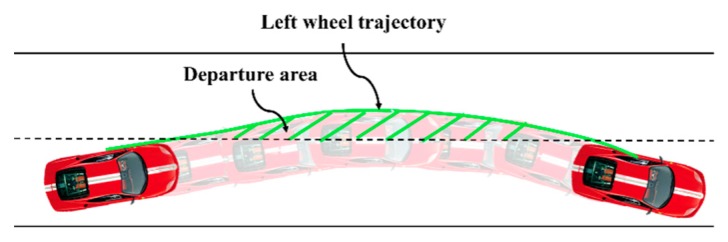
Illustration of departure area.

We use the predicted lateral distance to calculate departure area [29], which is utilized to evaluate the probability of PCSD when determining the driver’s ILCB. Departure area *S* is the area between the left (right) wheel trajectory and the lane boundary in the lane-departure event, as shown in Figure 5, and the formula is as follows:

(3) $$S=\int_{t_{start}}^{t_{end}}\left|D\left(t\right)\right|dt$$

where $t_{start}$ and $t_{end}$ are the starting and ending time of the departure behaviors, respectively.

Wenshuo Wang et al. [29] showed the statistical results of vehicles with/without the designed controller, including 200 left lane departure events and 200 right lane departure events that are both randomly produced from the stochastic lane departure model. From these experimental results, the lane departure behaviour is controllable when the departure area is less than 0.3. Correspondingly, the driver can safely guide the vehicle back to the original lane when the departure area is less than 0.3. Therefore, a formal description for deciding the occurrence of PCSD is performed as

(4) $$S=\{\begin{array}{l} S\leq 0.3,\;PCSD\\ S>0.3,\;Not\;PCSD \end{array}$$

## 3. Methods

### 3.1. Extreme Learning Residual Network

**Figure 6:**
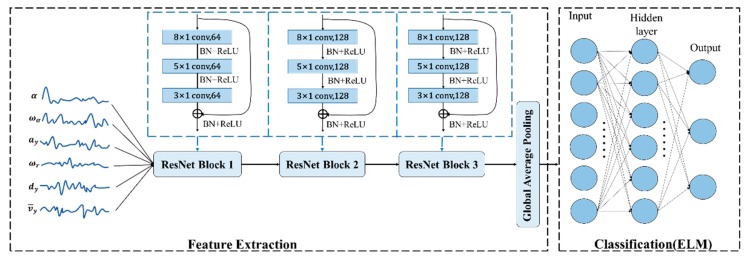
The Extreme Learning Residual Network (ELR-Net) architecture for driver behaviours classification. This architecture consists of six time-series inputs, three residual blocks, Global Average Pooling (GAP) layer, and Extreme Learning Machine (ELM). Each residual block consists of three convolutions, the lengths of the convolution filters are 8, 5, and 3. The number of filters for three residual blocks is 64, 128, and 128 respectively.

In this section, a deep learning framework is introduced to classify driver’s intention in the lane-departure event (ILCB or ULDB): ELR-Net. In the ELR-Net, ResNet [18] is chosen to extract the feature of time variable series as shown in Figure 6. The feature extraction part consists of three residual blocks, Global Average Pooling (GAP) and Class Activation Map (CAM). Global Average Pooling in the residual network is combined with CAM to determine the regions of the input variable sequence that have significant contributions to the classification. From Figure 6, ELM is used to multi-classify the extracted features in the classification part. 

#### 3.2.1. Activation Function in Residual Block

The ability of nonlinear modeling of neural networks can be improved by adding activation function to convolution neural networks in ResNet block activation layer. In this paper, ReLu (Rectified Linear Units) and BN (batch normalization) [30] are used as the activation function. The combination of ReLu and BN can ensure data stability and maintain the gradient without attenuation, thus accelerating the convergence speed of the network and improving the training speed of the network.

#### 3.2.2. Global Average Pooling Layer

Most network parameters are clustered in the full connection layer, which makes it easy for the network model to be over-fitted and reduces the generalization ability of the network. In this paper, the average pool layer replaces the full connection layer in the general network. Unlike the fully connected layer, the global average of each feature map is summarized to the last convolution output, allowing each feature graph to have one output. The average pool can greatly reduce the number of network parameters, avoid the over-fitting of the model, and accelerating the training speed and speed of the model. On the other hand, each feature mapping is equivalent to an output feature, which represents the characteristics of the output class.

#### 3.2.3. ELM for Multiclass Classification

ELM is suitable for feedforward neural networks with a single hidden layer and does not need to adjust the hidden layer. It has inherent advantages in dealing with multi-classification problems [16]. The output function of ELM is

(5) $$y\left(x\right)=\overset{L}{\underset{j=1}{\sum }}h_{j}\left(x_{i}\right)\beta_{j}=h\left(x\right)\beta$$

where h(x) is the output vector of the hidden layer; $\beta ={\left[\beta_{1,\ldots ,}\beta_{l}\right]}^{T}$, $\beta_{j}$ is the output vector weight of the hidden layer and the jth output node.

A classification matter of Extreme Learning Machine with multivariate time series input nodes and multiple output nodes can be expressed as

(6a) $$min\;L_{ELM}=\frac{1}{2}{\left\|\beta \right\|}^{2}+C\frac{1}{2}\overset{N}{\underset{i=1}{\sum }}{\left\|\xi_{i}\right\|}^{2}$$

(6b) $$s.t\;h\left(x_{i}\right)\beta =t_{i}^{T}-\xi_{i}^{T},\;i=1,\ldots \ldots ,N$$

where $\xi_{i}={\left[\xi_{i,1},\ldots ,\xi_{i,m}\right]}^{T}$ is the training error vector between the network output and the real value of the training sample $x_{i}$, C is a specified parameter and a trade-off between the distance of separation margin and the training error.

If the number of training samples is larger than the dimension of feature space, the result of solving output weight is as follows:

(7) $$\beta ={\left(1/C+H^{T}H\right)}^{-1}H^{T}T$$

The output function of the ELM classifier is

(8) $$y\left(x\right)=h\left(x\right)\beta =h\left(x\right){\left(1/C+H^{T}H\right)}^{-1}H^{T}T$$

The output function of the ELM classifier is a multi-classification problem, that is, six input variables and three output nodes. Let $y_{k}\left(x\right)$ denote the output function of the kth output node, i.e., $Y\left(x\right)={\left[y_{1}\left(x\right),y_{2}\left(x\right),y_{3}\left(x\right)\right]}^{T}$; then the predicted class label of sample x is

(9) $$label\left(x\right)=\arg\;\underset{i\in \left\{1,2,3\right\}}{\max}\;y_{i}\left(x\right)$$

### 3.2. ϵ-greedy LSTM

**Figure 7:**
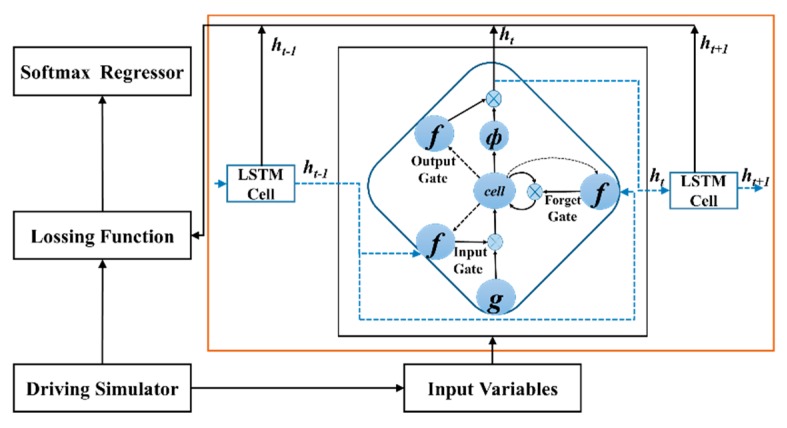
Structure of ϵ-greedy long short term memory (LSTM).

The ϵ-greedy LSTM mainly including four parts: Input Variables, LSTM, Lossing Function and Softmax Regressor, see Figure 7. In this algorithm, six variables extracted from the driver simulator are transferred to the network, then we are able to obtain the lateral distance value from the output of network. The performance of ϵ-greedy LSTM is evaluated in the next experimental part and its framework can extract effective time features very well.

LSTM is a special Recurrent Neural Network (RNN), which processes long-term information, solves long-term dependence, and prevents gradient losses or gradient explosions caused by serial inputs in transmission process [31]. Each LSTM unit has three gates: (a) forget gate $f_{t}$, (b) input gate $i_{t}$, and (c) output gate $o_{t}$. Two gates of LSTM control the contents of current cell state $c_{t}$: one is forget gate, which determines what information will be discarded from the previous cell state $c_{t-1}$; the other is input gate, which decides which new information will be stored in $c_{t}$. LSTM uses output gate to control the number of $c_{t}$ outputs to current output value $h_{t}$ of LSTM. For LSTM with input $x_{t}$ at time step t, three gates can be calculated as

(10a) $$i_{t}=sigmoid\left(x_{t}W_{x_{i},x_{t}}+h_{t-1}W_{h_{i},h_{t-1}}+b_{i}\right)$$

(10b) $$f_{t}=sigmoid\left(x_{t}W_{x_{f},x_{t}}+h_{t-1}W_{h_{f},h_{t-1}}+b_{f}\right)$$

(10c) $$o_{t}=sigmoid\left(x_{t}W_{x_{o},x_{t}}+h_{t-1}W_{h_{o},h_{t-1}}+b_{o}\right)$$

where $W_{i,j}$ is the weight matrices, and $b_{j}$ is the bias. The hidden state of RNN can be represented by h, and the unit update expression of LSTM is as follows:

(10d) $$C_{t}=C_{t-1}*f_{t}+i_{t}*tanh\left(x_{t}W_{x_{c},x_{t}}+h_{t-1}W_{h_{c},h_{t-1}}+b_{c}\right)$$

(10e) $$h_{t}=o_{t}*\tanh\left(C_{t}\right)$$

For *t* ∈ {1, 2, …, T}, the output value lateral distance D can be iteratively computed by

(11) $$D_{t}=W_{D_{t}}*LSTM\left(x_{t},h_{t-1};W\right)+b_{y}$$

In practical application, the loss function for the Softmax regression problem usually uses Mean Square Error (MSE) as a metric of network performance. The formula is as follows:

(12) $$MSE=1⁄N\overset{N}{\underset{i=1}{\sum }}{\left|\hat{D_{i}}-D_{i}\right|}^{2}$$

Greedy strategy derives the optimal value from the initial state of the problem through continuous greedy choice, which ensures that all data are trained to a certain extent and improves the learning accuracy of the neural network. Therefore, ϵ- greedy policy is used to improve the proposed LSTM model in this paper. To train the LSTM improved by the ϵ–greedy policy, the loss function of the proposed LSTM model is changed with the ϵ–greedy algorithm, which can be represented by

(13) $$L=F*\epsilon \left(\hat{D},D|\Theta \right)$$

where F is the loss function in LSTM; $\hat{D}$ is predicted value and D is observed value; Θ is the learning parameters of LSTM model; ϵ is the ratio of stochasticity in the greedy policy.

## 4. Analysis and Discussions of Experiment Result

### 4.1. Data Collection

**Figure 8:**
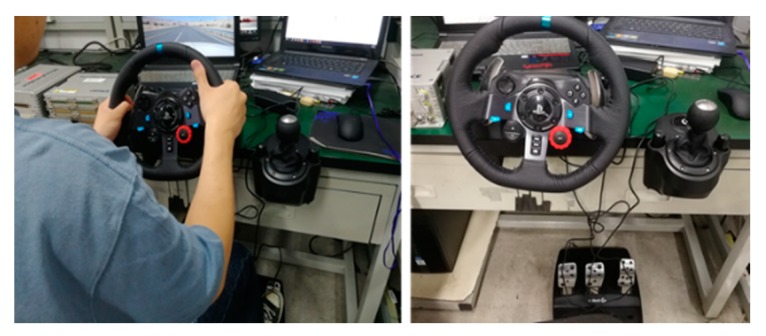
Driving Simulator.

The experimental data was collected from a driving simulator shown in Figure 8, which was equipped with some essential devices, including steering wheel angle sensor, steering motor and Brake & Accelerator pedal. In total, 21 volunteer participants (15 males and 6 females) were selected for experiment, as shown in Table 1. The collected sensor data included steering wheel angle, steering wheel angular velocity, lateral acceleration, yaw rate, lateral distance, and transverse average velocity. The driving data contains a reasonable amount of naturalistic ILCB, correction behaviors (DCB and PCSD), and ULDB (e.g., a lane departure behavior as the result of driver distraction and fatigue). In order to collect the data of lane departure behaviors as the result of driver distraction, we let the driver pick up the phone, chat or adjust the radio during the driving process. In the monotonous environment of the simulator, the driver can reach a very tired state after driving for more than 60 minutes [32]. In order to collect as much experimental data as possible from the driver’s fatigue, we selected the experiment within one hour after lunch, with each experiment lasting one hour. All driving data are synchronized and sampled at 10 Hz. Because the ϵ-greedy LSTM can accurately predict the lateral distance within 1 second, and the driver’s response time is usually within 1 second, the calculation time is increased by 1 second, so that the lane departure warning system can warn the driver in time.

### 4.2. Training and Test Set

Our dataset was split into five non-overlapping portions, where four portions of which were reserved for training and the remaining portion was utilized for testing. The mutuality between variables of $I_{DVR}$ does not take into consideration, thus each variable is an independent individual.

### 4.3. Classification Performance of ELR-Net

**Figure 9:**
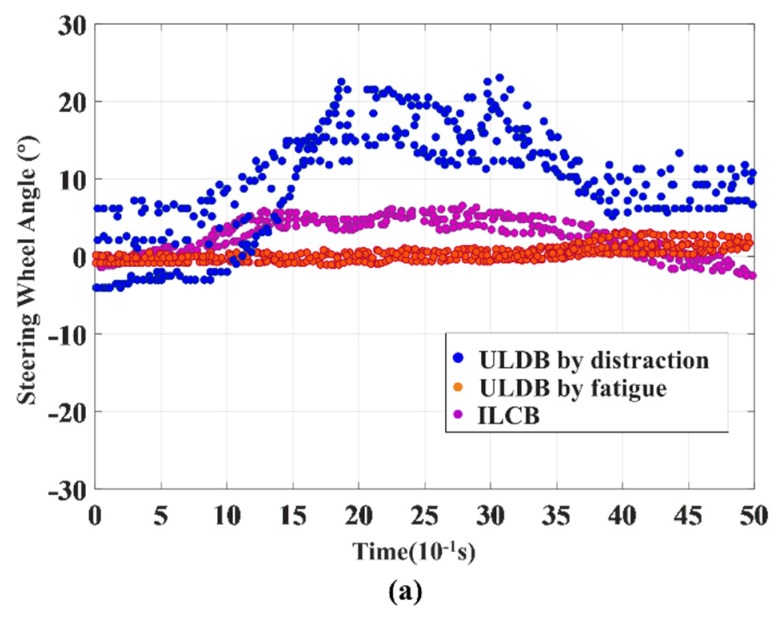

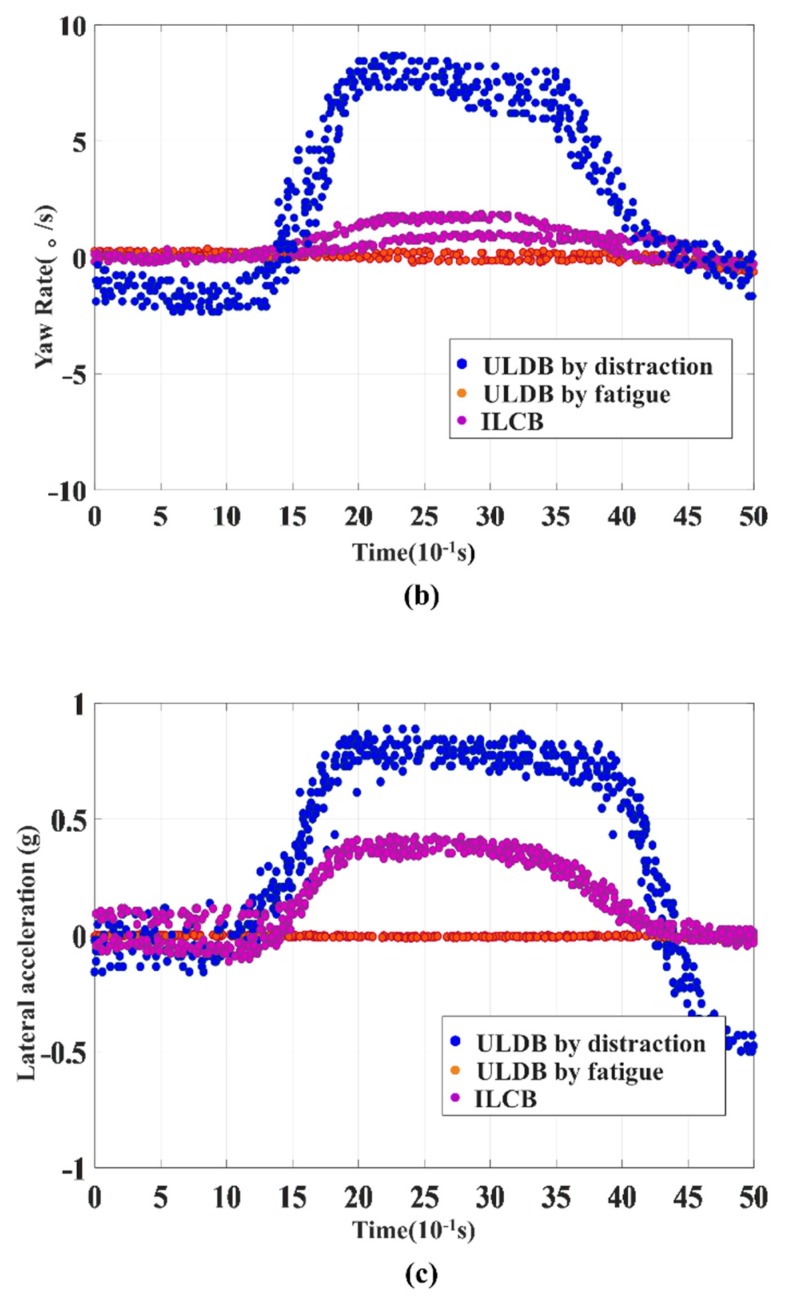
(**a**) Change curve of steering wheel angle under three behaviors; (**b**) Change curve of yaw rate under three behaviors; (**c**) Change curve of lateral acceleration under three behaviors.

In this section, we evaluated the classification performance of the proposed model in identifying between the initial episode of ILCB and ULDB. From the data collected by the driving simulator, we found that ILCB and ULDB have significant differences under various parameters. In addition, ULDB caused by distraction and fatigue also has significant differences under various parameters. In order to verify the accuracy of this conclusion, the steering wheel angle in Figure 9a, yaw rate in Figure 9b, and lateral acceleration in Figure 9c of the lane departure sample are randomly selected, as shown in Figure 9. From the variation curve of the 500 consecutive sampling points before the vehicle crosses the lane, the variation range of ULDB by distraction and ILCB is obviously larger than ULDB by fatigue, and the variation of parameters in the driver’s distraction state is greater than the intentional lane-changing, which is consistent with the actual situation. Specifically, the operating ability of the driver in the fatigue state is weakened and the vehicle response is relatively decreased. Therefore, the parameter variation of ULDB by fatigue in the Figure 9 is basically zero. In the distracted state, the driver often adjusts the vehicle, causing the vehicle to respond seriously.

Here, Classification Accuracy, Average Cost Loss [33,34] and Computation Time are used as indicators to assess ELR-Net performance. Average Cost Loss can be calculated by

(14) $$c\left(x,y\right)=\frac{1}{n}\overset{n}{\underset{i=1}{\sum }}P_{t}\left(\hat{y}|X_{t}^{i}\right)+\beta \times t$$

where *n* indicates the number of training time series, $P_{t}\left(\hat{y}|X_{t}^{i}\right)$ represents the misclassification probability that the time series $X_{t}^{i}$ is classified at time *t*, β×t expresses the delay cost of classification decision at time *t*, β is the penalty factor (predetermined constant). 

The calculation of the cost loss of the time series at each point in the experiment is computed according to the formula (14). When β = 0.0001, the experimental results of the experiments performed on MLP, FCN, ResNet, and ELR-Net are shown in Table 2.

**Figure 10:**
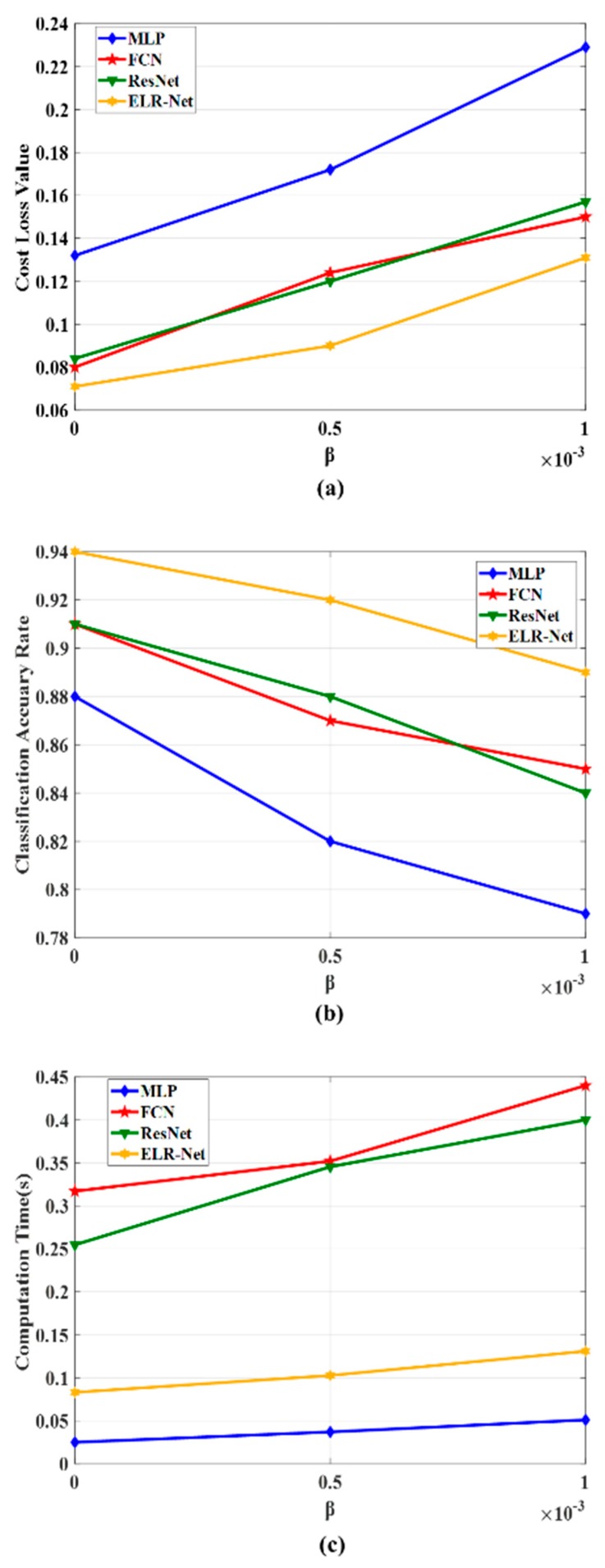
Performance comparison of MLP, FCN, ResNet, and ELR-Net, when β = 0.0001 β=0.0005 and β = 0.001.

In order to further prove the effectiveness of the algorithm, we take *β* = 0.0001, *β* = 0.0005, and *β* = 0.001 to experiment on MLP, FCN, ResNet, and ELR-Net; Cost Loss, Classification Accuracy, and Computation Time of the algorithm are shown in Figure 10. The classification result graph shows that in the case of the same *β* value, ELR-Net has a lower classification loss cost and higher classification accuracy than the other algorithms. In terms of computing time, ELR-Net is slightly longer than MLP. But the classification accuracy of the ELR-Net is much higher than MLP.

### 4.4. Prediction Performance of ϵ-Greedy LSTM

**Figure 11:**
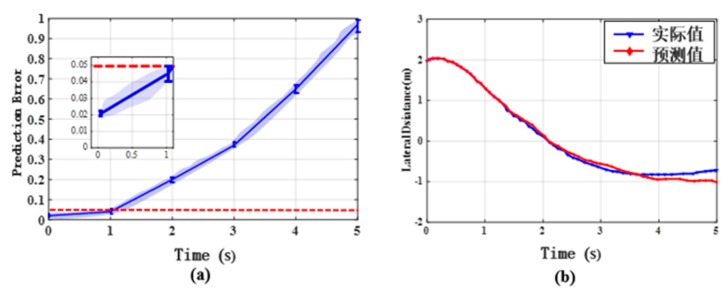
(**a**) Prediction errors of ϵ-greedy LSTM model as the prediction time increases from 0 s to 5 s; (**b**) prediction results of lateral distance in the five seconds.

In this section, we design additional experiments in order to evaluate the performance of ϵ-greedy LSTM. Effective prediction of lateral distance is crucial for judging the chance of PCSD. Figure 11a shows prediction errors of the proposed algorithm as the forecast time increases from 0 s to 5 s. The prediction error approximately increases from 0.02 to 0.96 and the error is 0.05 for a 1-s prediction time. Figure 11b gives the predicted and actual values of lateral distance in 5 seconds. It is easy to find that the prediction value and the actual value basically match in the 1-s period, and the accuracy of the prediction results decreases with the passage of time. Based on this result, ϵ-greedy LSTM can forecast distance in one second accurately. Additionally, we compare the proposed predictor with the conventional Linear approach and the basic LSTM approach. The Runtime and Root Mean Square Error (RMSE) are chosen as the criteria for assessing the performance of prediction result. Given a set of N size dataset, the RMSE can be calculated by

(15) $$RMSE=\sqrt{1/N\overset{N}{\underset{i=1}{\sum }}{\left(D-\hat{D}\right)}^{2}}$$

The above mentioned two criteria of the prediction results of Linear, LSTM, and ϵ-greedy LSTM models are demonstrated in Table 3. A notable observation is that the proposed method is able to reliably complete better prediction with considerable speed and accuracy. However, the linear model and traditional LSTM are not effective enough to obtain comfortable trade-offs in speed and accuracy, despite the three methods having roughly the same architectures.

### 4.5. Overall Performance of the Proposed LDWM

In this section, performance of the proposed LDWM is compared with other advanced methods including the basic TLC method [7] and TLC-DSPLS method [18]. The standard of performance comparison is false warning rate (FWR) and correct warning rate (CWR). Note that the false warning event is recorded as warning status when ILCB and/or PCSD occur, while the correct warning event is restricted as warning status when ULDB without PSCD occur during temporal interval of lane-departure identification. The rates are respectively computed by

(16a) $$FWR=\frac{false\;warning\;events}{all\;lane\;departure\;events}$$

(16b) $$CWR=\frac{correct\;warning\;events}{all\;lane\;departure\;events}$$

(16c) $$Warning\;Accuracy=\frac{correct\;identification\;events}{all\;lane\;crossing\;events}$$

**Figure 12:**
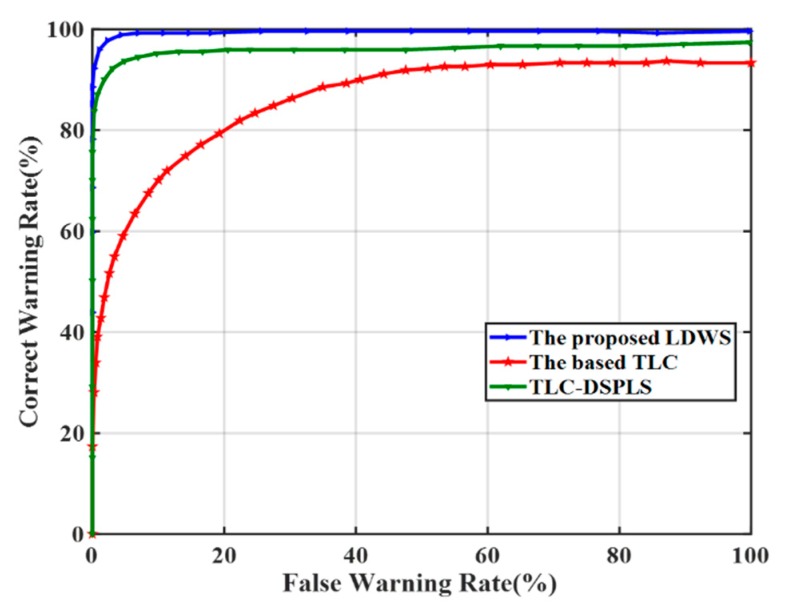
Comparison of False Warning Rate and Correct Warning Rate.

Figure 12 compares FWR and CWR in three different cases of the basic TLC, TLC-DSPLS, and the proposed LDWM. It can be seen from the diagram that the basic TLC algorithm has lowest warning accuracy, because of its inability to identify driver’s unconscious lane-departure behaviors. Due to the consideration of DCB, the FWR of TLC-DSPLS has great improvement compared to the basic TLC. Based on TLC-DSPLS, the proposed LDWM further figures out ILCB and PCSD and its largest FWR is about 1.2%. Table 4 shows the warning accuracy and its corresponding FWR and CWR. 

**Figure 13:**
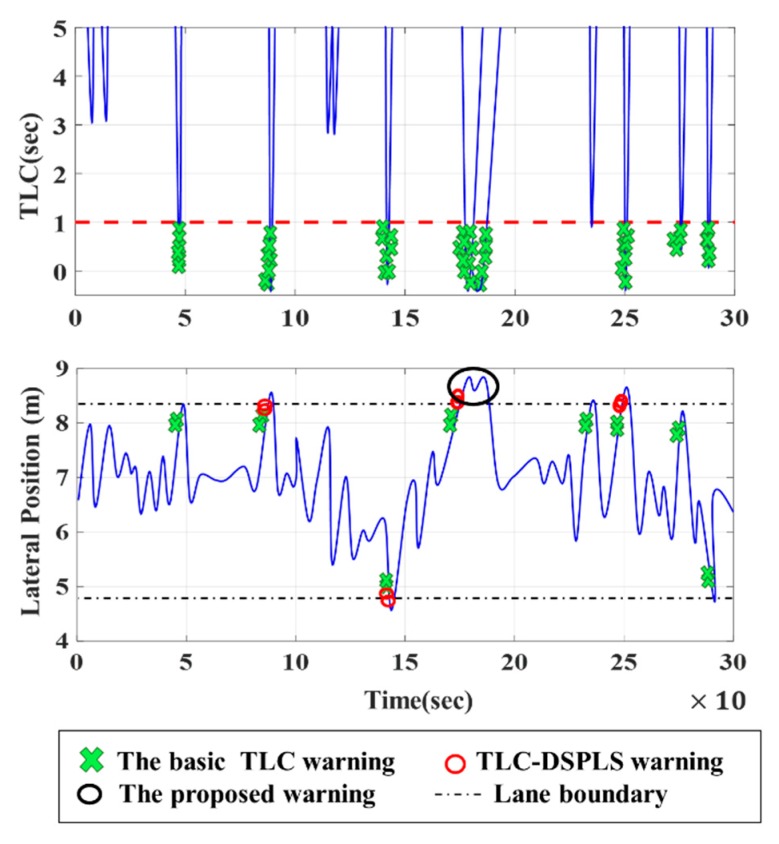
Comparison of alarm trigger position in three different warnings under TLC and lateral position. The basic TLC warning was triggered when the TLC value less than the threshold value (usually is defined as 1 s [18]).

Figure 13 gives an example comparing different LDWM employing the basic TLC warning, TLC-DSPLS warning, and the proposed warning. Although the TLC-based warning can warn drivers in time, the warnings are sometimes triggered at the inappropriate time. For the TLC-DSPLS warning, there are four false warnings in 86.1s, 141.7s, 175.0s, and 247.2s respectively. Worse, the basic TLC has eight false warnings severally in 44.4s, 83.3s, 144.2s, 172.2s, 230.6, 244.4s, 272.2, and 281.7s. As shown in Figure 13, the basic TLC and TLC-DSPLS easily trigger an alert when the driver is close to the lane boundary. The proposed warning can effectively reduce FAR by fully considering ILCB and PCSD.

## 5. Conclusions

In this paper, a promising technique to improve LDWM in terms of drivers’ acceptability is proposed and demonstrated by incorporating driver behavior information into the framework. Two novel network model (ELR-Net and ϵ-greedy LSTM) were presented and validated to identify driver behavioral intention. ELR-Net is a multi-time series classification network based on ResNet improvement, which is used to judge drivers’ real intentions in lane-changing events. ϵ-greedy LSTM effectively predicts the change of PCSD when the vehicle unconsciously departures from the lane. From the DVR characteristic, six variables are selected as the input of the network model, and lateral distance is used as the observation value to infer driver’s correction possibility. Last, to show the advantages of the proposed LDWM, we compare it with the basic TLC algorithm and TLC-DSPLS algorithm [35]. The results show that the proposed framework can precisely identify ILCB and PCSD, and reduce the FWR with trade-off for the CWR. Our future work will take more complicated real-world road environments into consideration, and apply the proposed strategy on more road scenes [36].

## Figures and Tables

**Table 1:** Statistical results of extracted driving data for all drivers.

Driver	# of Events	Total Time (min)	Average Time(s)	Driver	# of Events	Total Time (min)	Average Time(s)
1	564	207.04	22.02	12	584	204.31	20.97
2	550	197.17	21.52	13	608	212.09	20.93
3	313	109.88	21.07	14	569	200.07	21.09
4	285	107.38	22.62	15	540	190.66	21.17
5	311	108.37	20.88	16	542	197.72	21.89
6	470	170.35	21.75	17	454	166.10	21.95
7	360	130.44	21.78	18	488	177.23	21.78
8	278	96.93	20.88	19	473	173.72	22.01
9	268	94.43	21.15	20	513	189.03	22.11
10	320	110.78	20.79	21	423	153.63	21.78
11	605	211.68	20.98	Average	-	-	21.49

**Table 2:** Performance comparison of multilayer perceptron (MLP), fully convolutional networks (FCN), ResNet, and ELR-Net, when β = 0.0001.

Algorithm	Cost Loss	Classification Accuracy	Computation Time (s)
MLP	0.132	0.88	0.025
FCN	0.080	0.91	0.3171
ResNet	0.084	0.91	0.2547
ELR-Net	0.071	0.94	0.0831

**Table 3:** Prediction results for linear, LSTM, and *ϵ*-greedy LSTM.

Model	Detail	Loss Function	Runtime (/epoch)	RMSE
Linear	Dense layer + Softmax	MSE	nearly 8s	20.3094
LSTM	LSTM layer + Softmax	MSE	nearly 170s	11.5631
*ϵ*-greedy LSTM	LSTM layer + Softmax	MSE with *ϵ*-greedy	nearly 100s	5.5904

**Table 4:** Comparison of Warning Performance.

Algorithm	False Warning Rate (%)	Correct Warning Rate (%)	Warning Accuracy (%)
The basic TLC	14.1	75.2	87.7
TLC-DSPLS	2.3	96.1	97.4
The proposed LDWM	1.2	98.8	99.1
